# *N*-glycosylation Triggers a Dual Selection Pressure in Eukaryotic Secretory Proteins

**DOI:** 10.1038/s41598-017-09173-6

**Published:** 2017-08-18

**Authors:** Máximo Lopez Medus, Gabriela E. Gomez, Lucía F. Zacchi, Paula M. Couto, Carlos A. Labriola, María S. Labanda, Rodrigo Corti Bielsa, Eugenia M. Clérico, Benjamin L. Schulz, Julio J. Caramelo

**Affiliations:** 1 Fundación Instituto Leloir and Instituto de Investigaciones Bioquímicas de Buenos Aires (IIBBA-CONICET), 1405 Buenos Aires, Argentina; 2 Universidad de Buenos Aires. CONICET. Facultad de Farmacia y Bioquímica. Departamento de Química Biológica. Instituto de Química y Fisicoquímica Biológicas (IQUIFIB), 1113 Buenos Aires, Argentina; 3 School of Chemistry and Molecular Biosciences, The University of Queensland, Brisbane, QLD 4072 Australia; 4 Department of Biochemistry and Molecular Biology, Life Sciences Laboratories, University of Massachusetts, Amherst, MA 01003 USA; 5 Universidad de Buenos Aires. Facultad de Ciencias Exactas y Naturales. Departamento de Química Biológica, 1428 Buenos Aires, Argentina; 6 ARC Training Centre for Biopharmaceutical Innovation, The University of Queensland, St. Lucia, QLD 4072 Australia

## Abstract

Nearly one third of the eukaryotic proteome traverses the secretory pathway and most of these proteins are *N*-glycosylated in the lumen of the endoplasmic reticulum. *N*-glycans fulfill multiple structural and biological functions, and are crucial for productive folding of many glycoproteins. *N*-glycosylation involves the attachment of an oligosaccharide to selected asparagine residues in the sequence N-X-S/T (X ≠ P), a motif known as an *N*-glycosylation’sequon’. Mutations that create novel sequons can cause disease due to the destabilizing effect of a bulky *N*-glycan. Thus, an analogous process must have occurred during evolution, whenever ancestrally cytosolic proteins were recruited to the secretory pathway. Here, we show that during evolution *N*-glycosylation triggered a dual selection pressure on secretory pathway proteins: while sequons were positively selected in solvent exposed regions, they were almost completely eliminated from buried sites. This process is one of the sharpest evolutionary signatures of secretory pathway proteins, and was therefore critical for the evolution of an efficient secretory pathway.

## Introduction

Secretory pathway (SP) proteins enter the Endoplasmic Reticulum (ER), where they fold and become post-translationally modified by processes including *N*-glycosylation and disulfide bond formation^1, 2^. *N*-glycosylation is a highly conserved and key ER post-translational modification with multiple functions during and after protein folding^3^. The structural diversity of *N*-glycans allows for the protein’s participation in numerous recognition events on the cell surface. On the other hand, *N*-glycans can be considered as covalently attached chemical chaperones. *N*-glycans can promote the formation of secondary structural elements required for protein folding, increase the resistance towards proteolysis, and modify the half-life of secreted proteins^4–6^. *N*-glycans are also central for the activity of several chaperones and folding-assisting enzymes unique to the secretory pathway^2, 7^.

Approximately 80% of SP proteins are *N*-glycosylated at specific Asn residues, a process made dramatically more efficient by the presence of an *N*-glycosylation “sequon” (N-X-S/T, X ≠ P)^8^. The abundance of sequons has been shaped by the asymmetry of its coding sequence, in which loss of sequons is more likely due to mutations of the Asn residue, while gain of sequons is more probably attained by the generation of Ser/Thr downstream of a pre-existing Asn^9^. In higher eukaryotes, *N*-glycan attachment is performed by the STT3A and STT3B catalytic subunits of the membrane bound oligosaccharyl transferase complex (OST)^10, 11^. Complexes carrying STT3A preferentially recognize sequons co-translocationally and glycosylate acceptor sites as they enter the ER lumen, while STT3B-carrying complexes act post-translocationally and can modify sequons left vacant by STT3A^8^. This redundancy ensures a high occupancy level for most sequons. As the attachment of *N*-glycans confer the SP protein many beneficial properties, it can also interfere with the acquisition of the folded state, because of the *N*-glycan being attached to a sequon that is later buried in the protein, or because it is in a position that affects the protein’s folding pathway. The importance of the evolutionary fine-tuning of sequon localization is highlighted by the human genetic diseases associated with gain of *N*-glycosylation sequons^12^. In fact, many of these neo *N*-glycosylation sites are located in buried positions, leading to diverse diseases such as IFNγR2 deficiency^12^, hemophilia A^13^ and mucopolysaccharidosis IVA^14^.

## Results and Discussion

### Sequon frequency in integral membrane proteins depends on their topological orientation

**Figure 1:**
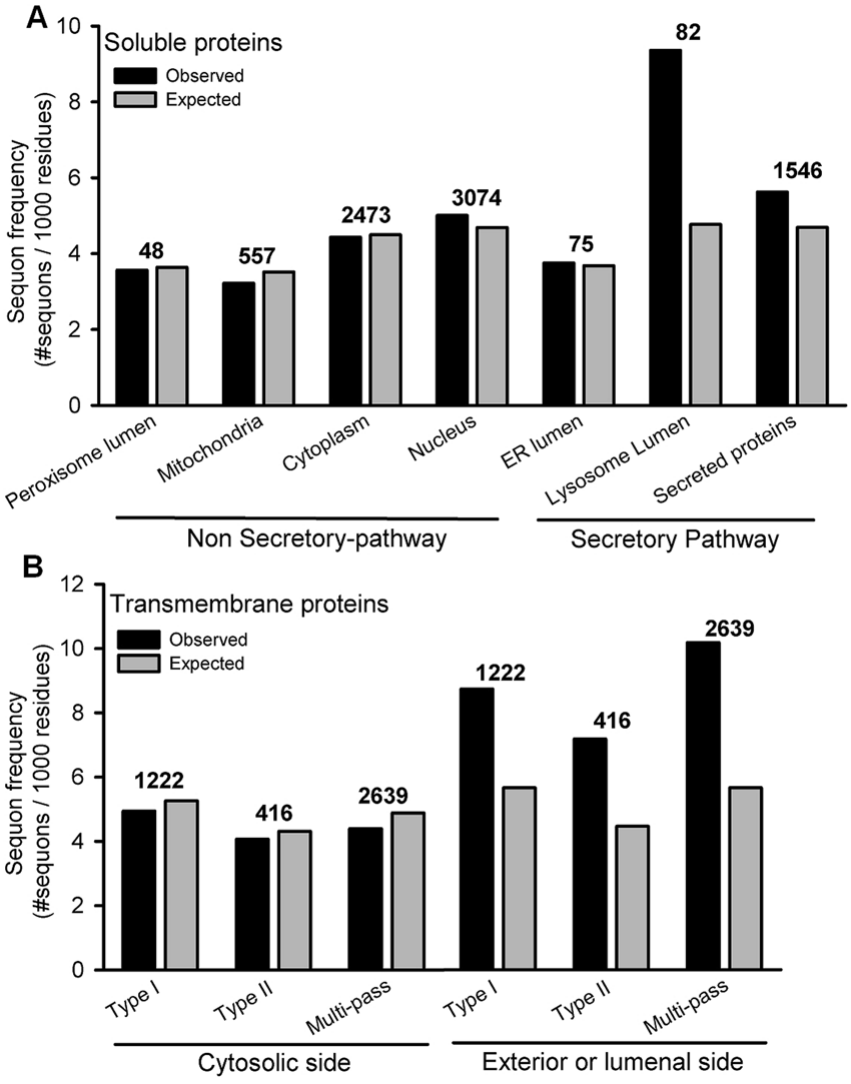
Sequon frequency in human proteins in different cellular compartments. Sequon frequency was measured for manually curated entries of human proteins in UNIPROT. (**A**) Soluble proteins in different cellular compartments. (**B**) Transmembrane proteins classified according to their orientation relative to the membrane. The number of proteins analyzed is indicated above the bars. The expected frequency was calculated from the amino acid abundance in each localization assuming a random distribution of amino acids within each protein (P_sequon_ = FREQ_Asn_ × (1-FREQ_Pro_) × (FREQ_Ser_ + FREQ_Thr_).

*N*-glycans play an essential role in the ER protein folding quality control system (QC)^2^. In fact, sequon abundance in SP proteins is higher than in non secretory-pathway (NSP) proteins, particularly in those organisms with QC based on *N*-glycans^15^. The higher sequon density displayed by SP proteins might depend on their final localization or their orientation relative to the membrane. To address this point we performed a statistical analysis of sequon abundance and localization in human proteins that are manually annotated in UNIPROT (Fig. 1). Not surprisingly, the observed sequon frequencies in soluble NSP proteins were very similar to expected values assuming a random distribution of amino acids, regardless of their final subcellular localization (Fig. 1A). In contrast, sequon frequencies in soluble SP proteins were higher than the expected values, and highly dependent on their subcellular localization. For instance, while ER soluble proteins do not display a significant difference between observed and expected frequencies, sequon abundance in lysosomal proteins was ~2-fold the expected value (Fig. 1A). This is a possible outcome of both, the known protection effect towards proteases exerted by *N*-glycans and the presence of mannose-6-phosphate lysosome targeting signals. More interestingly, sequon abundance in transmembrane proteins strongly depended on their orientation relative to the membrane (Fig. 1B). Sequon frequency in regions facing the lumen of secretory pathway organelles or the cell exterior was significantly higher than the expected value. On the contrary, expected and observed values were almost identical in those regions facing the cytosol (Fig. 1B). This is a clear example of differential selective pressure along the same gene, in this case dictated by the topological arrangement of the polypeptide. In addition, this differential sequon density could be employed as a constraint to better assign the topology of integral membrane proteins.

### Buried sequons can be occupied by OST

**Figure 2:**
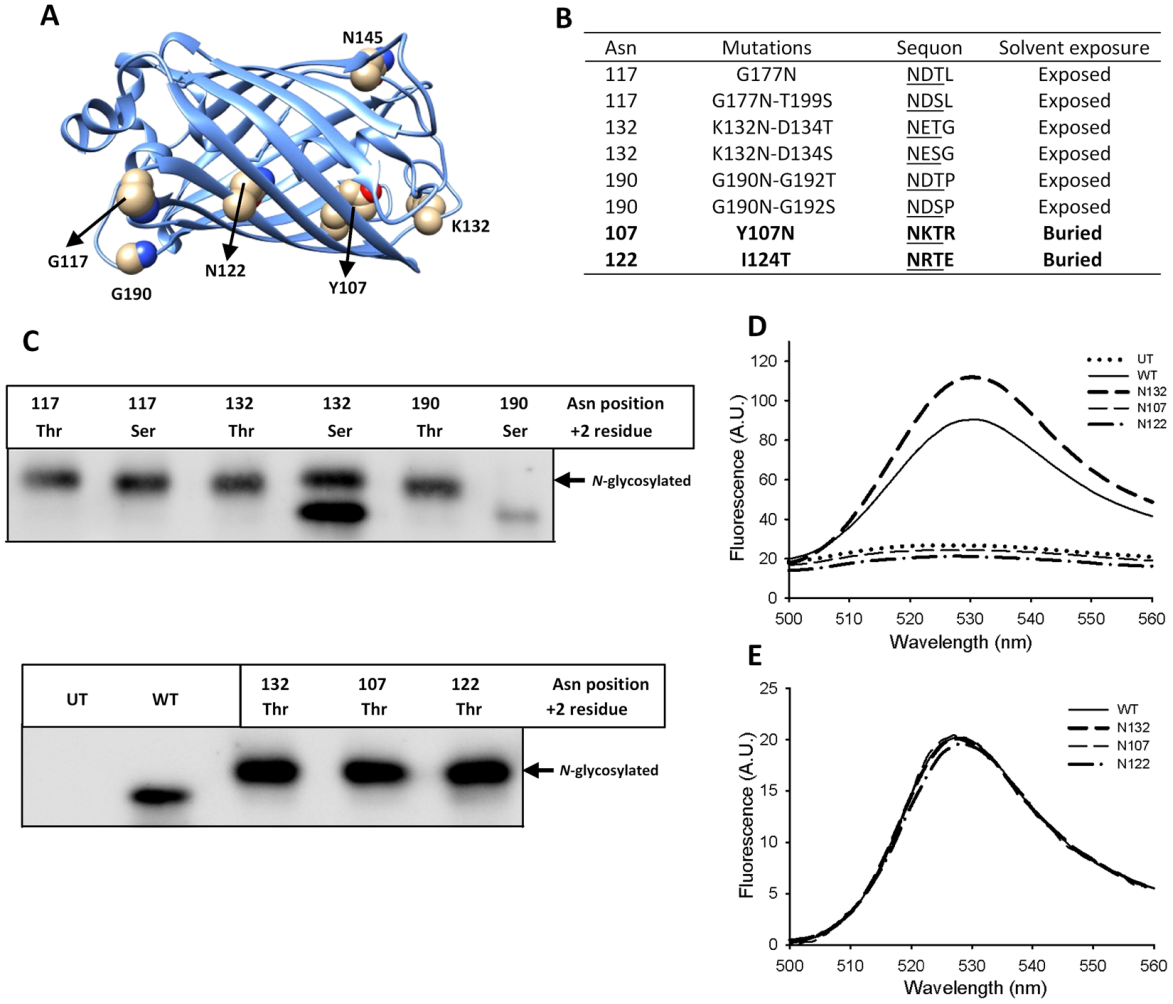
Buried sequons can be efficiently glycosylated. Yellow florescent protein (YFP) was fused with an N-terminal signal peptide, a C-terminal 3xHA epitope and a KDEL ER retention/retrieval signal. (**A**) Original residues in YFP (PDB code 1MYW) where Asn in sequons were located. Asn 145 shown in the image was previously used in the *N*-glycosylation sensor Glyc-ER-GFP^27^. (**B**) YFP *N*-glycosylation variants used in this study. Residues are numbered as in the mature protein after signal peptide cleavage. (**C**) anti-HA western blot analysis of COS-7 cells expressing variants YFPs. (UT: untransfected cells, WT: wild type non-glycosylatable YFP). Original gels are shown in Supplementary Fig. S5. (**D**) Emission spectra of transfected COS-7 cells resuspended in lysis buffer. Fluorescence of cells expressing YFP with glycosylation at buried sites sites (variants N107 or N122) is indistinguishable from the fluorescence of untransfected cells. **(E)** Emission spectra of proteins purified from *E*. *coli* (without *N*-glycosylation).

It is estimated that approximately 35% of all sequons in SP proteins are partially occupied or remain unglycosylated^16^. Therefore, many SP proteins display a mixture of glycoforms with partial sequon occupancy, a feature known as *N*-glycosylation macroheterogeneity. Factors controlling this phenomenon are partially understood. Coevolution of enzyme and protein substrates is a requirement for efficient glycosylation, but enzyme:substrate complementarity may not always be optimal^17, 18^. Enzyme isoforms can also exist with different protein substrate specificities. For instance, yeast OST exists as two isoforms incorporating either Ost3 or Ost6 subunits with different protein substrate specificities^19, 20^, and sequons near the C-terminal end of mammalian proteins are poorly recognized by STT3A-OST and are mainly glycosylated by STT3B-OST^21^. In addition, sequons with Thr at the +2 position are generally more efficiently *N*-glycosylated than those with Ser, and the occupation of the latter is more dependent on residues at positions +1 and +3 in the sequon^22–24^. We experimentally confirmed this using an ER-localized YFP. Sequons placed in surface exposed loops of this protein were more efficiently occupied when they had Thr instead of Ser at the +2 position (Fig. 2A,C). Previous analysis of occupied sequons showed that *N*-glycans are typically located in solvent-exposed positions, more frequently in turns of folded polypeptides at boundaries between secondary structural elements^25^. In some cases the *N*-linked GlcNAc is in close contact with nearby amino acids, filling a cleft on the protein’s surface. In principle, OST activity is blind to the final output of the folding process and it can glycosylate sequons that would be present in buried positions of properly folded unglycosylated variants. Indeed, the folding kinetics of carboxypeptidase Y can modulate the occupancy of artificially introduced ´buried´ sequons^26^. We introduced glycosylation sequons in our model YFP glycoprotein with the Asn facing the interior of the protein in the folded state (Fig. 2A). These structurally buried sequons were efficiently glycosylated, but the resulting YFPs did not fluoresce (Fig. 2C–E). A previously described GFP-based *N*-glycosylation sensor takes advantage of a similar principle^27^. Its fluorescence is lost when the sequon is occupied, although in this case the lateral chain of the Asn is exposed, pointing outside the GFP barrel (N145 in Fig. 2A). Proteins in which buried sequons were inappropriately glycosylated would likely be recognized as misfolded, retained in the ER by the folding quality control systems, and aggregated or retrotranslocated to the cytosol for degradation^3, 28^.

### Buried sequons suffered a negative selection pressure

**Figure 3:**
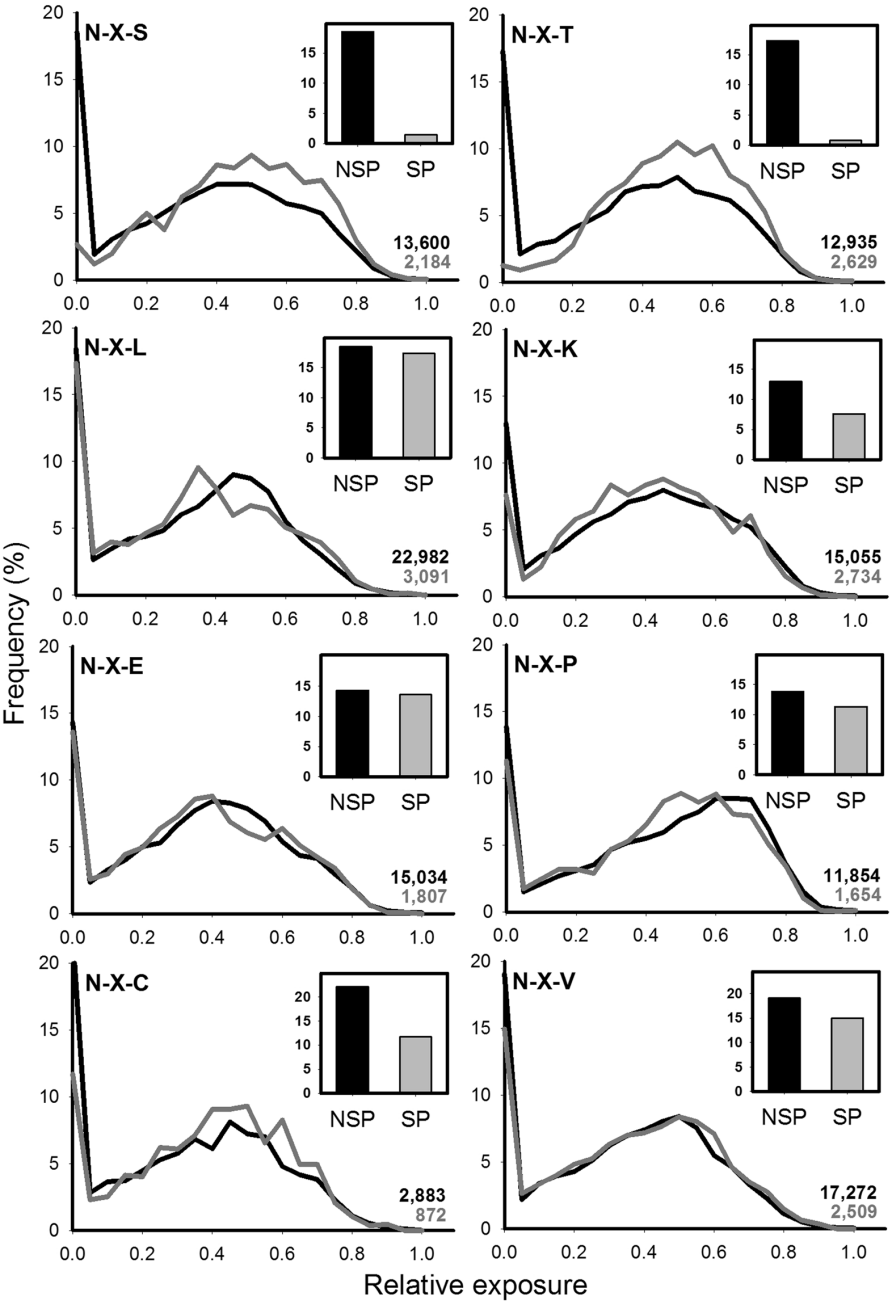
Surface exposure distribution of Asn residues in SP and in NSP proteins. The main graphs show the distribution of exposure of Asn residues in the selected tripeptides for NSP (black lines) and SP proteins (gray lines), where X can be any residue. The total number of Asn analyzed for NSP and SP proteins is indicated in black and gray, respectively. Inset show the percentage of buried Asn in each curve (exposure values below <5%).

We hypothesized that during evolution *N*-glycosylation in eukaryotes triggered a negative selection pressure that led to the disappearance of buried sequons present in the folded state of SP proteins. To test this idea we measured the relative surface exposure of amino acids of SP and NSP proteins in a non-redundant set of the PDB. The subcellular locations of these proteins were assigned according to their UNIPROT annotations and curated manually. The dataset had 4,165 and 26,349 SP and NSP proteins, respectively (Supplementary File 1). Surface exposure was calculated with MSMS using a spherical probe of radius 3 Å, approximately the size of a monosaccharide^25^. A residue was considered buried when its relative surface accessibility was less than 5%. Overall, the relative surface exposure distribution for the 20 amino acids in SP and NSP proteins were very similar (Supplementary Fig. S1). The proportion of buried residues in both sets of proteins was also similar, and correlated well with their hydrophobicity except for Cys, a consequence of the burying of many disulfide bridges (Supplementary Fig. S2). The main difference between SP and NSP proteins was observed for Ser and Thr, both displaying a lower frequency in SP proteins. This observation is likely a consequence of the role of these residues in *N*-glycosylation sequons. We next calculated the relative exposure of each amino acid (A1) in all possible tripeptides A1-X-A3, where X could be any residue. Here again SP and NSP proteins showed similar exposure profiles (Fig. 3). Remarkably, the main exceptions were the tripeptides Asn-X-Ser and Asn-X-Thr, whose exposure profiles exhibited a sharp difference in the proportion of buried Asn in SP vs NSP proteins, indicating that buried Asn in the context of sequons are rare in SP proteins (p-values < 0.001, Pearson’s chi-squared test). The profiles of tripeptides Asn-X-Cys were similar for SP and NSP proteins (Fig. 3), suggesting that *N*-glycosylation of such contexts is a rare event that does not alter the global trend.

**Figure 4:**
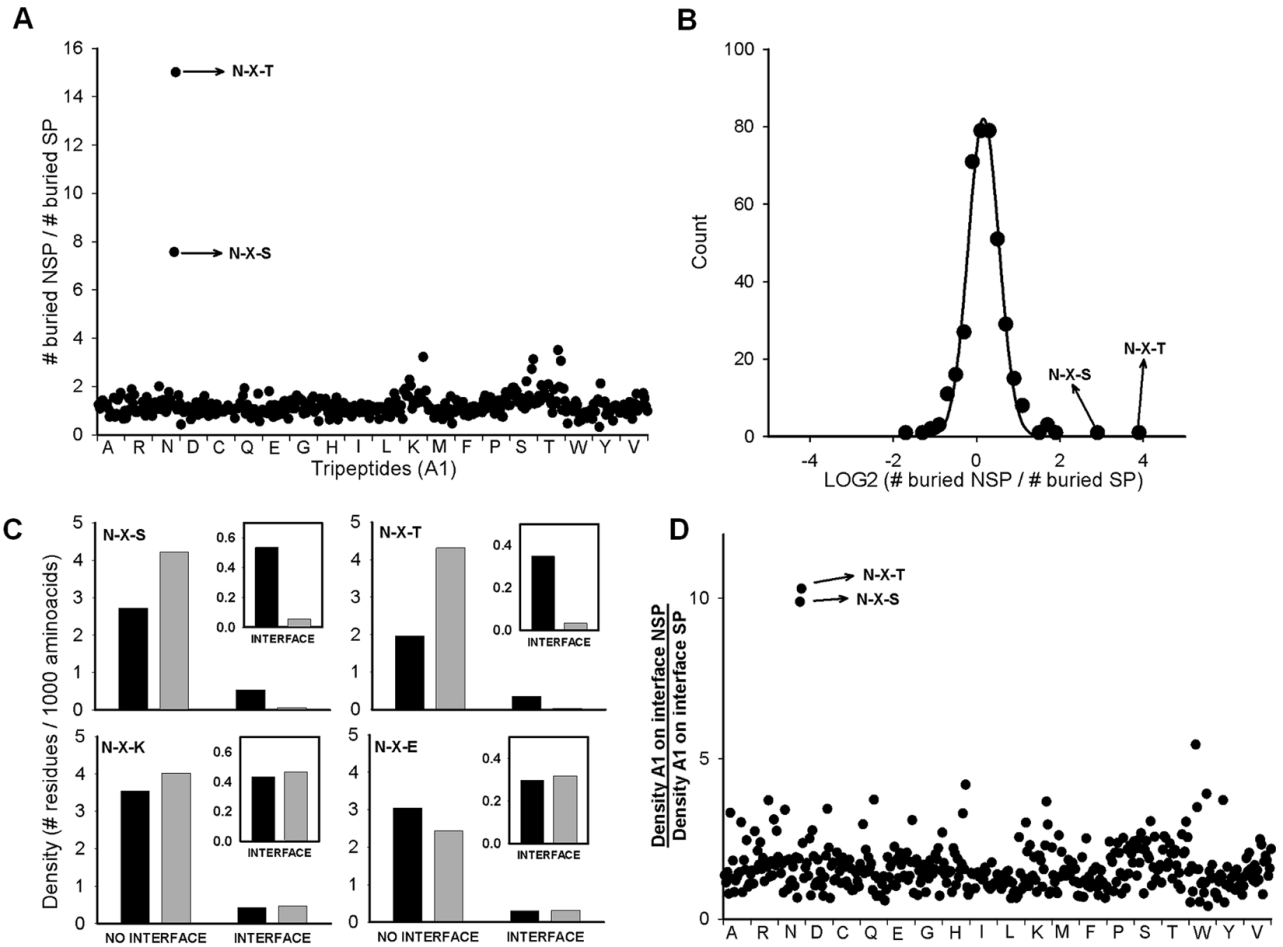
Sequence characteristics of buried residues in secretory and non-secretory pathway proteins. (**A**) Ratio of the number of buried A1 residues in A1-X-A3 tripeptides in NSP compared with SP proteins. Residue A1 of each tripeptide is shown in the x axis. (**B**) Log2 distribution of the ratios from panel A. A Gaussian curve was fitted to the data. (**C**) Frequency of exposed Asn in the indicated tripeptides located at protein-protein interfaces or elsewhere on protein surfaces. NSP and SP proteins are in black and gray, respectively. A total of 1,209,862 and 147,995 residues were analyzed for NSP and SP protein complexes, respectively. Inset: zoom of density values for residues at the interface. (**D**) Ratio of A1 residue densities at interfaces of NSP compared with SP proteins for all A1-X-A3. In panels **A** and **D** each dot represents one of the 400 possible tripeptides arranged in alphabetic order (Ala-X-Ala, Ala-X-Arg, …, Val-X-Val).

A global comparison of amino acid exposure was performed by calculating the ratio of buried A1 residues between SP and NSP proteins for the 400 possible combinations of tripeptides A1-X-A3. Most tripeptides analyzed yielded a ratio near one, indicating no difference between the exposure of the selected residues for SP and NSP proteins. The most prominent exceptions to this result were the tripeptides Asn-X-Ser and Asn-X-Thr (Fig. 4A,B). While in NSP proteins approximately 17% of Asn in these contexts appeared in buried positions, this number drastically dropped for SP proteins (2.7% for Asn-X-Ser and 1.2% for Asn-X-Thr). A similar but smaller trend was previously found using a sequence-based algorithm that predicts amino acid solvent accessibility^29^. That study found that 24.4% of *N*-glycosylation sequons are located at positions classified as solvent inaccessible. In our non-redundant dataset of SP proteins there were 57 and 31 buried Asn in the tripeptides Asn-X-Ser and Asn-X-Thr, respectively (Supplementary Table S1). Nine of these Asn are in fact *N*-glycosylated in the solved structures. These glycosylated residues were located in pockets or clefts that were poorly accessed by the spherical probe employed and were classified as buried. Twenty one of these buried Asn residues corresponded to tripeptides where X was Pro, which are not *N*-glycosylation sequons. In addition, many buried sequons were in non-efficient contexts: 4 were placed near a transmembrane domain and 17 had residues at +1 or +3 positions that negatively affect OST activity^22–24^. This last point should be considered with caution. For instance, even though Asp at +1 position and Pro at the +3 position are likely detrimental for sequon occupancy, the YFP mutant with an NDTP sequon (mutations G190N-G192T) is efficiently *N*-glycosylated, while the analogous mutant with NDSP sequon (mutations G190N-G192S) remains vacant (Fig. 2C). Interestingly, three buried Asn in Asn-Asp-Ser sequons (PDB codes: 1IE4, 1TFP and 2QPF) belonged to transthyretins. Normally, these cryptic sites are not occupied. Prolonged exposure of these sites during delayed protein folding triggers their modification by the STT3B-dependent posttranslational mechanism, which in turn leads to the degradation of these proteins^30^. Human transthyretin (PDB code 2WQA) was also analyzed in the original dataset. Its NDS sequon is slightly more exposed than its counterparts, and for this reason it was classified as exposed. On the other hand, 12 sequons were placed near the C-terminal end of the protein, a location that impairs cotranslocational *N*-glycosylation. Nevertheless, in some cases these sequons can by recognized by the STT3B^21^. Together, this showed that buried Asn-X-Ser and Asn-X-Thr sequences in SP proteins are rare, and many of those that do exist are in contexts that could impair efficient glycosylation by OST. These results show that the scarcity of buried Asn in glycosylation sequons is a distinctive feature of SP proteins. However, not all sequons are glycosylated with equal efficiency by OST. It was expected that buried sequons that would be efficiently glycosylated would disappear during evolution. A different situation arises for sequons that are inefficiently glycosylated by OST. In this case, only a small proportion of the pool of protein carrying such sequons would be glycosylated. Any protein that was glycosylated at these sequons would likely be degraded, so the mature protein would essentially only be present in the non-glycosylated form. Since the majority of the total synthesized pool of such proteins would be functional, it might be expected that these sequences would have persisted during evolution. However, we found that this scenario was rare, since almost all buried sequons disappeared, including those that are predicted to be inefficiently glycosylated. The efficiency of this evolutionary selection process is remarkable.

### Buried sequons are rare at protein-protein interfaces

As *N*-glycosylation sequons were rare in buried locations in folded proteins, we hypothesized that they would also be disfavored at protein-protein interfaces. We tested this by exploring the occurrence of sequons at interfaces in a set of structures of protein complexes from the PDB. In total, we analyzed 5,179 and 709 complexes of NSP and SP proteins, respectively. Amino acid surface exposure in the biologically active complexes and in their isolated subunits was calculated using MSMS. Residues displaying an accessibility difference larger than 90% between these values were assigned as buried at protein-protein interfaces. We then compared the frequency of all tripeptide sequences at protein-protein interfaces in NSP and SP proteins. The frequency of most tripeptides was similar at interfaces for NSP and SP proteins (Fig. 4C). As observed for protein core-buried sequons, the most drastic differences in tripeptide occurrence at protein-protein interfaces were the tripeptides Asn-X-Ser and Asn-X-Thr (p-values < 0.001, Pearson’s chi-squared test) (Fig. 4C,D). Asn in these tripeptides were essentially absent at protein-protein interfaces of SP proteins and, conversely, appeared with a higher frequency than that in NSP proteins outside protein-protein interfaces. Of a total 1,274 sequons, there were only 13 Asn-X-Ser and 7 Asn-X-Thr buried at protein-protein interfaces of SP proteins (Supplementary Table S2). Seven of these had Pro at the +1 position, 2 had a suboptimal sequon (Asp or Glu at +1 position), and 3 were near the protein’s C-terminus. One of the buried sequons belonged to the mannose-binding lectin concanavalin A from *Canavalia ensiformis*. This sequon is generated through a post-translational polypeptide ligation process that takes place after its passage through the ER, and is therefore not a substrate of the OST^31^. Taken together, these analyses showed that buried Asn in glycosylation sequons are also unusual at protein-protein interfaces of SP protein complexes.

### Sequon loss in BiP was necessary for its biological activity

**Figure 5:**
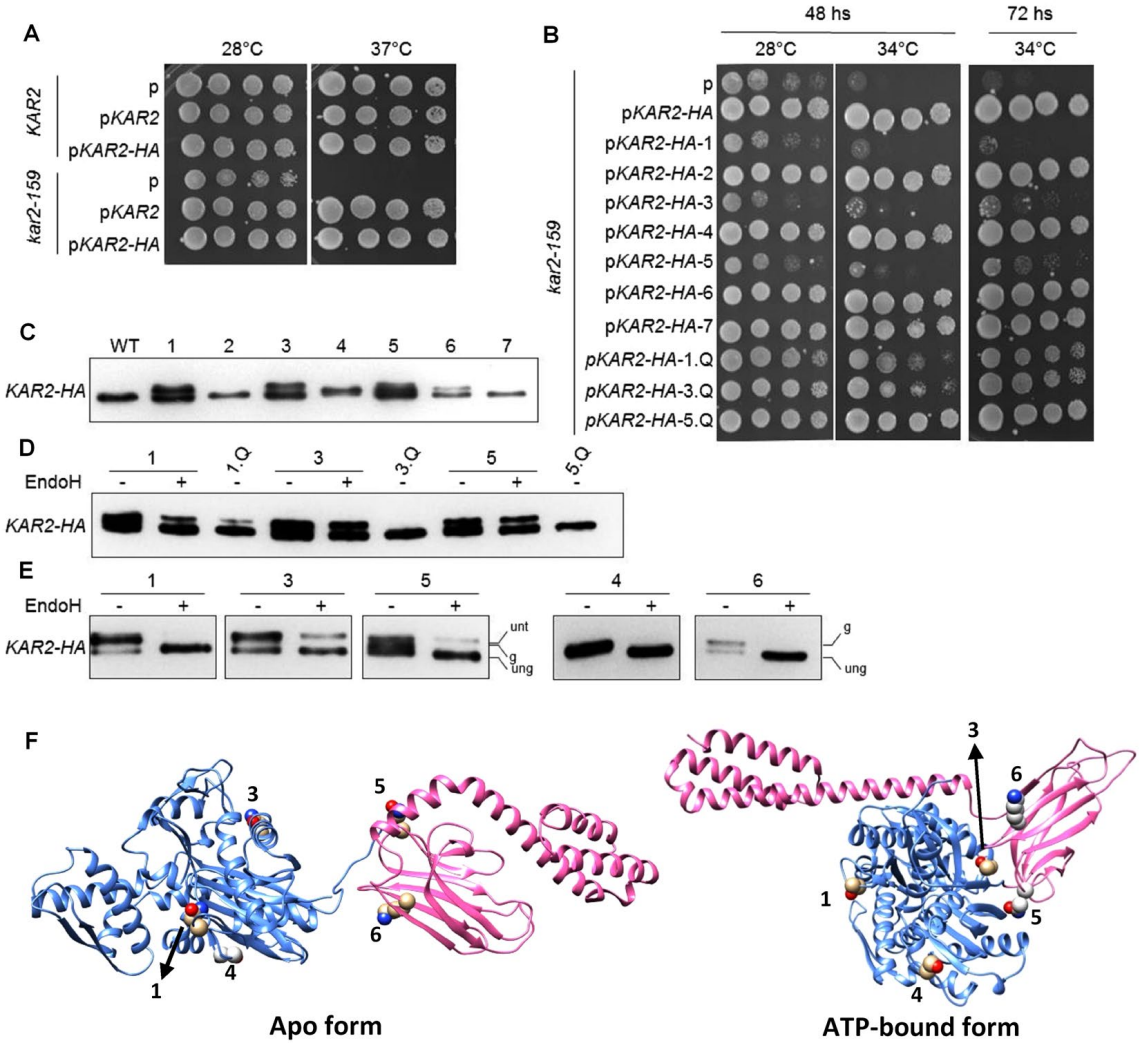
Introducing *N*-glycosylation sites in yeast Kar2 severely impairs its function. The wild-type yeast strain *KAR2* and the temperature sensitive mutant *kar2-159* were transformed with an empty vector (p), or vectors for the expression of Kar2, Kar2-HA, variant Kar2-HA introducing sequons 1 through 7 (see text for details), or Kar2-HA sequon-revertants 1.Q, 3.Q, or 5.Q, as indicated. In sequon revertants, the introduced sequons encoding Asn were replaced by the codon encoding Gln. (**A**) Liquid cultures of transformed KAR2 and kar2-159 strains or (**B**) kar2-159 strains transformed with KAR2-HA glycosylation variants were grown overnight, serially diluted, spotted onto SC-URA plates and grown at the indicated temperatures and times. (**C**) anti-HA western blot from kar2-159 transformed with wild type KAR2-HA or glycosylation variants 1–7. (**D**) and (**E**) Protein extracts with or without EndoH treatment, separated through SDS-PAGE, and blotted with anti-HA antibody. (ung) is unglycosylated, (g) is glycosylated, (unt) is untranslocated. (**F**) Cartoon structures of Kar2 showing the location of introduced glycosylation sequons that were glycosylated. N-terminal and C-terminal domains are depicted in blue and pink ribbons, respectively.

Our data suggested that sequons in buried positions in mature folded proteins or protein-protein interfaces had been subject to a negative selection process in proteins that entered the SP. We experimentally tested this using the Hsp70 family of chaperones, which has members in diverse subcellular compartments. Hsp70s prevent protein aggregation by recognizing hydrophobic stretches exposed on their client proteins. These chaperones consist of an N-terminal ATPase domain, and a C-terminal substrate binding domain followed by a short non-structured segment. In the ADP-bound or nucleotide-free (‘apo’) forms of the ATPase domain, the C-terminal domain has a closed conformation, and both domains can move relative to one another with few restrictions. In this conformation substrate binding or release is very slow. The exchange of ADP by ATP facilitated by nucleotide exchange factors triggers a dramatic conformational change that brings both domains into close contact and stabilizes an open conformation of the C-terminal domain^32^. Substrate binding to this state is fast and activates the ATPase domain facilitated by J-domain proteins, shifting the conformational equilibrium of the C-terminal domain back to the substrate-bound closed conformation. The Hsp70 ER homologue (BiP) is one of the most abundant proteins in this subcellular compartment^33^. Many SP proteins interact with BiP as they enter the ER^34^. In addition, BiP has a central role in sensing ER stress to initiate the unfolding protein response^35^. The common ancestor of the eukaryotic Hsp70s is the cytosolic Hsp70 from bacteria, where *N*-glycosylation is absent. Bacterial Hsp70s typically have numerous sequons (Supplementary Fig. S3). Similarly, eukaryotic Hsp70s located in subcellular locations other than the ER also typically contain several sequons. This is in sharp contrast with most ER Hsp70s, which have almost no sequons. Three notable exceptions to this pattern are BiPs from *Theileria Parva*, *Theileria annulata* and *Encephalitozoon hellem*, which have several sequons. Consistently, these protozoans are among the few eukaryotes that lack *N*-glycosylation^15^. Another exception is BiP from *Plasmodium falciparum*, which has three sequons with Ser at the +2 position. However, *P*. *falciparum* transfers a very short GlcNAc_2_ *N*-glycan, which could be more easily accommodated than the bulkier *N*-glycans transferred by most eukaryotes^36^. These observations suggest a model in which selective pressure led to the elimination of sequons in BiP in order to allow for its biological role. We tested this hypothesis using Kar2, the orthologue of BiP in *Saccharomyces cerevisiae*. The cytosolic Hsp70s in this yeast (Ssa1 and Ssa4) have several sequons, which are absent in Kar2 (Supplementary Fig. S4). We individually introduced these sequons into a functional Kar2 fused to a C-terminal HA tag (Fig. 5A)^37^. Asn-Xaa-Thr was used for all introduced sequons to maximize their occupancy (Supplementary Table S3). These variant Kar2 proteins were expressed under the control of the native *KAR2* promoter in a strain carrying the thermosensitive variant Kar2-159. The Kar2-159 protein has a mutation in the ATPase domain (G417S) that affects protein translocation into the ER at restrictive temperatures^38^. Sequons in variants 1, 3, 4, 5 and 6 showed various degrees of occupancy, while sequons in variants 2 and 7 were not detectably modified (Fig. 5C,D). In addition, some variants had a minor impairment in their translocation into the ER, as revealed by the remaining slow migrating band after removing *N*-glycans with endoglycosidase H (EndoH) (Fig. 5E). While wt Kar2 could rescue the growth defect at 34 °C, the efficiently glycosylated variants 1, 3 and 5 were unable to rescue the deleterious phenotype (Fig. 5B). This effect was due to functional defects resulting from the presence of *N*-glycans rather than from the other altered amino acids in the introduced sequons, as the effect was reverted by changing the Asn in the introduced sequons to Gln (Fig. 5D). The sequon in variant 1 is partially occluded in the ATP binding site of the N-terminal domain of Kar2 (Fig. 5F). A glycan in that position would likely inhibit the enzymatic activity of the chaperone. Sequons in variants 3 and 5 are located at the interdomain docking surface in the ATP-bound form of the chaperone (Fig. 5F)^39^. A bulky *N*-glycan in this region would preclude domain docking thus abolishing the essential function of Kar2. Partial occupancy of sequons in variants 4 and 6 did not affect the activity of Kar2. These sequons are located in exposed sites in both the apo and the ATP-bound forms of the chaperone (Fig. 5F). Together, these results show that *N*-glycosylation of BiP is generally detrimental, and suggests that the evolutionary driven loss of *N*-glycosylation sequons was necessary to ensure the proper biological function of this crucial ER chaperone.

### Gain of sequons in buried sites is associated with human genetic diseases

An independent physiological example of the importance of the evolutionary fine-tuning of sequon localization are the human genetic diseases associated with gain of *N*-glycosylation sequons. These neo *N*-glycosylation sites account for 1.4% of human genetic diseases^12^ with 238 known mutations in 151 genes that encode proteins with signal peptides or transmembrane helices. In order to better understand the molecular basis of these pathologies, we analyzed the structural localization of these anomalous sequons. Eighty of these neo sequons occur in 39 proteins with known structure. Inspection of these proteins revealed that 37 of these sequons (46%) are located in buried positions (Supplementary File 2). This proportion is likely to be still higher, since many of the remaining exposed sequons could be located at protein-protein interfaces of stable or transient functional complexes whose structure is not yet known.

In summary, we have explored the evolutionary consequences of protein *N*-glycosylation on secretory proteins. Our bioinformatic, biochemical, and biological analyses suggest that during evolution *N*-glycosylation triggered a dual selection process in eukaryotic secretory proteins: sites became more abundant in exposed regions and were essentially eliminated from buried locations. This process occurred with remarkable efficiency, because *N*-glycosylation sites that are tolerated in cytoplasmic proteins severely impact the function of paralogous proteins in the secretory pathway. This redistribution of the location of *N*-glycosylation sites is one of the sharpest evolutionary signatures of proteins that enter the secretory pathway, and was therefore critical for the evolution of an efficient secretory pathway in eukaryotic cells.

## Methods

### Bioinformatics analysis

Analysis of sequon frequency in human proteins was performed with the manually curated entries of UNIPROT, using their assigned subcellular localization. The orientation of transmembrane proteins was derived from their annotated topological domains. For the analysis of amino acid exposition, a non-redundant set of the PDB was generated with CD-HIT using a 95% identity cutoff (http://weizhongli-lab.org/cd-hit). The non-redundant dataset had 26,349 and 4,165 NSP and SP proteins, respectively. Protein localization was first assigned according to their annotation in UNIPROT, and all assignments were manually curated. Signal peptide prediction was performed with SignalIP. (www.cbs.dtu.dk/services/SignalP/) and cellular localization with TargetP 1.1 (www.cbs.dtu.dk/services/TargetP)^40^. All heteroatoms and hydrogens were deleted from the corresponding PDB files. Amino acid surface exposure was calculated using MSMS (mgltools.scripps.edu/packages/MSMS) with a probe of radius 3 Å. Sequon frequency at protein-protein interfaces was determined for all biological complexes in the dataset using the biological units of the PDB. This dataset had 5,179 and 709 complexes of NSP and SP proteins, respectively. Residue exposure was calculated in the biological complexes and in their isolated subunits using MSMS. Residues displaying an accessibility difference bigger than 90% between these values were assigned as buried at protein-protein interfaces. Statistical analysis of buried Asn within sequons was performed with a Pearson´s chi-squared test implemented in Sigma Plot 11.0, where the null hypothesis is the frequency of buried Asn in NSP proteins. Protein graphics were generated using Chimera 1.10.2^41^. Structural models of *S*. *cerevisiae* BiP were generated with Modeller 9.15 implemented in Chimera 1.10.2 using pdbs 2KHO and 5E84 as templates^42^.

### Introduction of sequons in YFP

Primers and plasmids employed are listed in Supplementary Tables S4 and S5, respectively. YFP fused to the signal peptide of calreticulin, a C-terminal 3xHA epitope and a KDEL ER retention/retrieval sequence was employed as starting material. This was cloned in a pcDNA3.1 vector (ThermoFisher) using the restriction sites KpnI and XbaI. Mutations were introduced using an overlapping PCR approach^43^. First, two PCR products were generated using generic external primers (SP6 and T7) and internal, overlapping primers containing the desired point mutation, using KOD Hot Start DNA Polymerase (Millipore). The following mutagenic primer pairs were employed: JJC228-JJC229 (G117N), JJC230-JJC231 (G117N-T119S), JJC232-JJC233 (K132N-D134T), JJC233-JJC235 (K132N-D134S), JJC236-JJC237 (G190N-G192T), JJC238-JJC239 (G190N-G192S), JJC340-JJC341 (Y107N) and JJC344-JJC345 (I124T).

### COS-7 cell transfection

COS-7 cells from ATCC (CRL-1651) were grown at 37 °C, 5% CO2 in Dulbecco’s modified Eagle’s medium supplemented with 10% FBS. Contamination with *Mycoplasma* was tested with MycoFluor™ (ThermoFisher). Cells were regularly passaged to maintain exponential growth in P100 plates. 24 hours before transfection cells were seeded into 24-well plates at 85,000 cells/well. Transfection of 1 µg pcDNA3.1 vector/well with YFP or mutant DNA was carried out with Lipofectamine 2000 (Life Technologies) as described by the manufacturer for adherent cell lines. Subsequent measurements (fluorescence, immunocytochemistry) were performed 24 hours after transfection.

### Western blot of YFPs

COS-7 cells transfected in 24-well plates were washed once with 200 µL PBS/well. Cells were lysed in 100 µL of lysis buffer (25 mM Tris-Cl, 150 mM NaCl, 1% Tritón X-100, 1 mM EDTA, pH 7.5) with protease inhibitors for one hour on ice. Then, adherent cells were scrapped off the well using a plastic cell scraper. Cellular lysates were boiled for 5 min in Laemmli’s sample buffer and were subjected to 12.5% SDS-polyacrylamide gel electrophoresis (PAGE) followed by Western blot analysis. The proteins were electrophoretically transferred from the gel onto PVDF membranes using Tris-glycine buffer. To block nonspecific binding, membranes were incubated with 5% nonfat milk in Tris buffer for 1 hour at room temperature. The blots were incubated with rat anti-HA (1:10000 dilution) primary antibody (Roche, 11867423001) plus 5% nonfat milk in TBS at 4 °C overnight. After washing with TBS buffer, the blots were incubated with HRP-conjugated goat anti-rat IgG (1:5000 dilution) secondary antibody (Sigma-Aldrich, A9037) plus 5% nonfat milk in TBS buffer for 1 hour at room temperature. After washing with TBS, ECL detection (GE Healthcare) and autoradiography was employed to identify specific protein bands.

### Expression and purification of YFP

The genes encoding wild-type (wt) or sequon mutants YFP were cloned into the pET-22b(+) vector (Novagen). The plasmids were transformed into the *E*. *coli* strain BL26. The cells were cultured to 0.6 OD600 nm in Luria-Bertani liquid medium with 200 µg/mL ampicillin at 37 °C. Protein expression was induced overnight at 20 °C by adding 1 mM isopropyl-β-D-1-thiogalactoside. The cells were collected by centrifugation (4,000 rpm, 15 min, 4 °C), resuspended in buffer A (50 mM Tris, 150 mM NaCl, pH 8) and lysed by sonication. To remove insoluble components, the lysates were centrifuged at 4 °C and 10,000 rpm for 30 min. YFPs were isolated from the supernatants by nickel-affinity chromatography (GE Healthcare) and eluted with buffer B (100 mM Tris-HCl, 500 mM NaCl, 150 mM imidazole, pH 8.0). The eluates were precipitated by adding (NH_4_)_2_SO_4_ (70% w/v), centrifuged for 50 min at 35,000 rpm, and resuspended in 50 mM Tris-HCl, 150 mM NaCl, pH 8.0. The proteins were further purified by size-exclusion chromatography in a Superdex 200 10/300 GL column (GE Healthcare) using PBS buffer. The purified YFPs were frozen with liquid nitrogen and stored at −20 °C.

### Fluorescence spectroscopy

Emission spectra of COS-7 cell lysates or YFP purified from *E*. *coli* cultures were performed at 25 °C on a Jasco FP-6500 spectrofluorimeter equipped with a thermostated cell. A 3 mm path cuvette sealed with Teflon caps was used. The spectral slit-widths were set to 3 nm for each monochromator. The excitation wavelength was set to 480 nm and emission spectra were collected in the range 500–560 nm.

### Introduction of sequons in Kar2

A PCR fragment containing the *KAR2* gene (YJL034W, open reading frame (ORF) +499 bp of promoter region, and 300 bp of terminator region) was PCR amplified with KOD Hot Start from strain BY4741 DNA using primers LZBS15 and LZBS16. The PCR fragment was digested with BamHI and SacI, ligated into pRS416 digested with the same enzymes, and transformed into *E*. *coli* Top10 chemical competent cells to generate plasmid Ec316. A C-terminal HA tag was added to Kar2, before the HDEL sequence^35, 44, 45^, using primers LZBS20 and LZBS21 following previously published protocols^46^, to generate plasmid Ec317. Point mutations to introduce sequons into Kar2 were performed as follows. Vector Ec335 containing sequon 1 was constructed by first PCR amplifying *KAR2* from plasmid Ec317 using the mutagenic primer pairs JJC558 and LZBS21 and JJC559 and LZBS15, followed by an overlapping PCR of the resulting PCR fragments using primers LZBS15 and LZBS21^43^. The resulting fragment was digested with BamHI and XbaI and ligated into Ec317 BamHI/XbaI digested vector. Vector Ec330 containing sequon 2 was constructed using primer pairs JJC643 and JJC644 as described in reference 42. Vectors Ec318-326 containing sequons 3–7 were constructed by amplifying the entire Ec317 vector with the mutagenic primer pairs JJC562 and JJC563 (sequon 3, Ec321), JJC564 and JJC565 (sequon 4, Ec326), JJC566 and JJC567 (sequon 5, Ec318), JJC568 and JJC569 (sequon 6, Ec319), and JJC570 and JJC571 (sequon 7, Ec320). To eliminate select introduced sequons from vectors, we made Asn to Gln mutations as follows. To eliminate sequon 1 from Ec335 and sequon 3 from Ec321, the same strategy to construct Ec335 was used, but employing primer pairs LZBS26 and LZBS27 to make Ec381, and LZBS28 and LZBS29 to make Ec382. To eliminate the sequon from Ec318 and generate Ec383, the entire Ec317 vector was amplified using mutagenic primers LZBS30 and LZBS31. The entire ORF in all cloned material was sequenced.

### Yeast strains and growth conditions

Yeast strains used in this study are described in Supplementary Table S6 were generously provided by Jeffrey Brodsky (University of Pittsburgh, USA). All strains were grown at 28 °C (except when specified) on YPD (1% yeast extract, 2% peptone, 2% dextrose) or synthetic complete (SC) media lacking uracil for auxotrophic selection. Transformation of yeast strains was performed using the standard protocol for the lithium acetate/PEG3350 method, except that heat shock was performed at 37 °C. To test the heat sensitivity of the different mutants, overnight SC-URA cultures of single colonies were serially 1:5 diluted (after normalization to 2 OD600 nm), and spotted onto SC-URA plates. Plates were incubated at 28 °C or 34 °C for 48 h. To test for protein expression, 2 OD600nm of cells from the overnight cultures were pelleted, frozen, and processed as described below.

### Yeast protein extraction and western blot

Yeast protein extracts were prepared for western blotting as previously described^47^. Samples resolved by SDS-PAGE were transferred to nitrocellulose membranes and blotted using the following antibodies: monoclonal mouse anti-HA (1:2000 dilution, ThermoFisher, 32–6700) and HRP-conjugated horse anti-mouse IgG (1:2000 dilution, Cell Signaling, 7076S). Western blots were developed with Supersignal West Pico Chemiluminescent Substrate (Pierce). To monitor the acquisition of *N*-linked glycosylation, whole cell protein extracts were digested with 1 U of Endoglycosidase H (New England Biolabs) overnight at 37 °C. Samples were resolved by SDS-PAGE and used for western blotting, as described above.

## Electronic supplementary material

Supplementary Information

Dataset 1

Dataset 2

## References

[1] Braakman I, Hebert DN (2013). Protein folding in the endoplasmic reticulum. Cold Spring Harb. Perspect. Biol..

[2] Caramelo JJ, Parodi AJ (2015). A sweet code for glycoprotein folding. FEBS Lett..

[3] Hebert DN, Lamriben L, Powers ET, Kelly JW (2014). The intrinsic and extrinsic effects of N-linked glycans on glycoproteostasis. Nat. Chem. Biol..

[4] Imperiali B, O’Connor SE (1999). Effect of *N*-linked glycosylation on glycopeptide and glycoprotein structure. Curr. Opin. Chem. Biol..

[5] Mitra N, Sinha S, Ramya TN, Surolia A (2006). *N*-linked oligosaccharides as outfitters for glycoprotein folding, form and function. Trends Biochem. Sci..

[6] Shental-Bechor D, Levy Y (2009). Folding of glycoproteins: toward understanding the biophysics of the glycosylation code. Curr. Opin. Struct. Biol..

[7] Hebert DN, Molinari M (2012). Flagging and docking: dual roles for *N*-glycans in protein quality control and cellular proteostasis. Trends Biochem. Sci..

[8] Shrimal S, Cherepanova NA, Gilmore R (2015). Cotranslational and posttranslocational *N*-glycosylation of proteins in the endoplasmic reticulum. Sem. Cell Dev. Biol..

[9] Williams R (2014). Encoding asymmetry of the *N*-glycosylation motif facilitates glycoprotein evolution. Plos ONE.

[10] Kelleher DJ, Gilmore R (2006). An evolving view of the eukaryotic oligosaccharyltransferase. Glycobiology.

[11] Mohorko E, Glockshuber R, Aebi M (2011). Oligosaccharyltransferase: the central enzyme of *N*-linked protein glycosylation. J. Inherit. Metab. Dis..

[12] Vogt G (2005). Gains of glycosylation comprise an unexpectedly large group of pathogenic mutations. Nature Gen..

[13] Lin SW, Lin SR, Shen MC (1993). Characterization of genetic defects of hemophilia A in patients of Chinese origin. Genomics.

[14] Bunge S (1997). Identification of 31 novel mutations in the N-acetylgalactosamine-6-sulfatase gene reveals excessive allelic heterogeneity among patients with Morquio A syndrome. Hum. Mutat..

[15] Cui J, Smith T, Robbins PW, Samuelson J (2009). Darwinian selection for sites of Asn-linked glycosylation in phylogenetically disparate eukaryotes and viruses. Proc. Natl. Acad. Sci. USA.

[16] Apweiler R, Hermjakob H, Sharon N (1999). On the frequency of protein glycosylation, as deduced from analysis of the SWISS-PROT database. Bioch. Biophys. Acta.

[17] Zacchi LF, Schulz BL (2016). *N*-glycoprotein macroheterogeneity: biological implications and proteomic characterization. Glycoconj. J..

[18] Schulz, B. L. Beyond the Sequon: Sites of *N*-Glycosylation. *InTech*., doi:10.5772/50260 (2012).

[19] Schulz BL, Aebi M (2009). Analysis of glycosylation site occupancy reveals a role for Ost3p and Ost6p in site-specific *N*-glycosylation efficiency. Mol. Cell. Prot..

[20] Schulz BL (2009). Oxidoreductase activity of oligosaccharyltransferase subunits Ost3p and Ost6p defines site-specific glycosylation efficiency. Proc. Natl. Acad. Sci. USA.

[21] Shrimal S, Trueman SF, Gilmore R (2013). Extreme C-terminal sites are posttranslocationally glycosylated by the STT3B isoform of the OST. J. Cell Biol..

[22] Kasturi L, Eshleman JR, Wunner WH, Shakin-Eshleman SH (1995). The hydroxy amino acid in an Asn-X-Ser/Thr sequon can influence *N*-linked core glycosylation efficiency and the level of expression of a cell surface glycoprotein. J. Biol. Chem..

[23] Shakin-Eshleman SH, Spitalnik SL, Kasturi L (1996). The amino acid at the X position of an Asn-X-Ser sequon is an important determinant of *N*-linked core-glycosylation efficiency. J. Biol. Chem..

[24] Mellquist JL, Kasturi L, Spitalnik SL, Shakin-Eshleman SH (1998). The amino acid following an asn-X-Ser/Thr sequon is an important determinant of *N*-linked core glycosylation efficiency. Biochemistry.

[25] Petrescu AJ, Milac AL, Petrescu SM, Dwek RA, Wormald MR (2004). Statistical analysis of the protein environment of *N*-glycosylation sites: implications for occupancy, structure, and folding. Glycobiology.

[26] Holst B, Bruun AW, Kielland-Brandt MC, Winther JR (1996). Competition between folding and glycosylation in the endoplasmic reticulum. EMBO J..

[27] Losfeld ME, Soncin F, Ng BG, Singec I, Freeze HH (2012). A sensitive green fluorescent protein biomarker of *N*-glycosylation site occupancy. FASEB J..

[28] Benyair R, Ogen-Shtern N, Lederkremer GZ (2015). Glycan regulation of ER-associated degradation through compartmentalization. Semin. Cell Dev. Biol..

[29] Park C, Zhang J (2011). Genome-wide evolutionary conservation of *N*-glycosylation sites. Mol. Biol. Evol..

[30] Sato T (2012). STT3B-dependent posttranslational *N*-glycosylation as a surveillance system for secretory protein. Mol. Cell.

[31] Carrington DM, Auffret A, Hanke DE (1985). Polypeptide ligation occurs during post-translational modification of concanavalin A. Nature.

[32] Kityk R, Kopp J, Sinning I, Mayer MP (2012). Structure and dynamics of the ATP-bound open conformation of Hsp70 chaperones. Mol. Cell.

[33] Gidalevitz T, Stevens F, Argon Y (2013). Orchestration of secretory protein folding by ER chaperones. Bioch. Biophys. Acta.

[34] Vembar SS, Jonikas MC, Hendershot LM, Weissman JS, Brodsky JL (2010). J domain co-chaperone specificity defines the role of BiP during protein translocation. J. Biol. Chem..

[35] Gardner BM, Pincus D, Gotthardt K, Gallagher CM, Walter P (2013). Endoplasmic reticulum stress sensing in the unfolded protein response. Cold Spring Harbor Persp. Biol..

[36] Bushkin GG (2010). Suggestive evidence for Darwinian Selection against asparagine-linked glycans of *Plasmodium falciparum* and *Toxoplasma gondii*. Eukaryotic Cell.

[37] Murray PJ, Watowich SS, Lodish HF, Young RA, Hilton DJ (1995). Epitope tagging of the human endoplasmic reticulum HSP70 protein, BiP, to facilitate analysis of BiP–substrate interactions. Analytical Bioch..

[38] Kimata Y (2003). Genetic evidence for a role of BiP/Kar2 that regulates Ire1 in response to accumulation of unfolded proteins. Mol. Biol. Cell.

[39] Yang J, Nune M, Zong Y, Zhou L, Liu Q (2015). Close and Allosteric Opening of the Polypeptide-Binding Site in a Human Hsp70 Chaperone BiP. Structure.

[40] Emanuelsson O, Brunak S, von Heijne G, Nielsen H (2007). Locating proteins in the cell using TargetP, SignalP and related tools. Nature Prot..

[41] Pettersen EF (2004). UCSF Chimera–a visualization system for exploratory research and analysis. J. Comp. Chem..

[42] Sali A, Blundell TL (1993). Comparative protein modelling by satisfaction of spatial restraints. J. Mol. Biol..

[43] Ho SN, Hunt HD, Horton RM, Pullen JK, Pease LR (1989). Site-directed mutagenesis by overlap extension using the polymerase chain reaction. Gene.

[44] Donoso G, Herzog V, Schmitz A (2005). Misfolded BiP is degraded by a proteasome-independent endoplasmic-reticulum-associated degradation pathway. Biochem. J..

[45] Wang J, Pareja KA, Kaiser CA, Sevier CS (2014). Redox signaling via the molecular chaperone BiP protects cells against endoplasmic reticulum-derived oxidative stress. eLife.

[46] Imai Y, Matsushima Y, Sugimura T, Terada M (1991). A simple and rapid method for generating a deletion by PCR. Nucl. Acids Res..

[47] Zacchi LF (2014). The BiP molecular chaperone plays multiple roles during the biogenesis of torsinA, an AAA+ ATPase associated with the neurological disease early-onset torsion dystonia. J. Biol. Chem..

