# Supplementary material for: N-glycosylation Triggers a Dual Selection Pressure in Eukaryotic Secretory Proteins
Source: Sci Rep. 2017 Aug 18;7:8788. doi: 10.1038/s41598-017-09173-6 (PMC5562741; doi:10.1038/s41598-017-09173-6)
Supplement: Supplementary file 1 — Supplementary Information [file 41598_2017_9173_MOESM1_ESM.pdf]

Supplementary Information

N-glycosylation Triggers a Dual Selection Pressure in Eukaryotic Secretory Proteins

Máximo Lopez Medus[1,*], Gabriela E. Gomez[2,*], Lucía F. Zacchi[3,*,†], Paula M. Couto[1,*], Carlos A. Labriola[1], María S. Labanda[1], Rodrigo Corti Bielsa[1], Eugenia M. Clérico[4], Benjamin L. Schulz[3] & Julio J. Caramelo[1,5]

[1]Fundación Instituto Leloir and Instituto de Investigaciones Bioquímicas de Buenos Aires (IIBBA-CONICET), 1405 Buenos Aires, Argentina. [2]Universidad de Buenos Aires. CONICET. Facultad de Farmacia y Bioquímica. Departamento de Química Biológica. Instituto de Química y Fisicoquímica Biológicas (IQUIFIB), 1113 Buenos Aires, Argentina. [3]School of Chemistry and Molecular Biosciences, The University of Queensland, QLD 4072, Australia. [4]Department of Biochemistry and Molecular Biology, Life Sciences Laboratories, University of Massachusetts, Amherst MA 01003, USA. [5]Universidad de Buenos Aires. Facultad de Ciencias Exactas y Naturales. Departamento de Química Biológica, 1428 Buenos Aires, Argentina. [†]Present address: ARC Training Centre for Biopharmaceutical Innovation, The University of Queensland, St. Lucia, QLD 4072, Australia. [*]These authors contributed equally to this work. Correspondence and requests for materials should be addressed to J.J.C. (email: jcaramelo@leloir.org.ar)

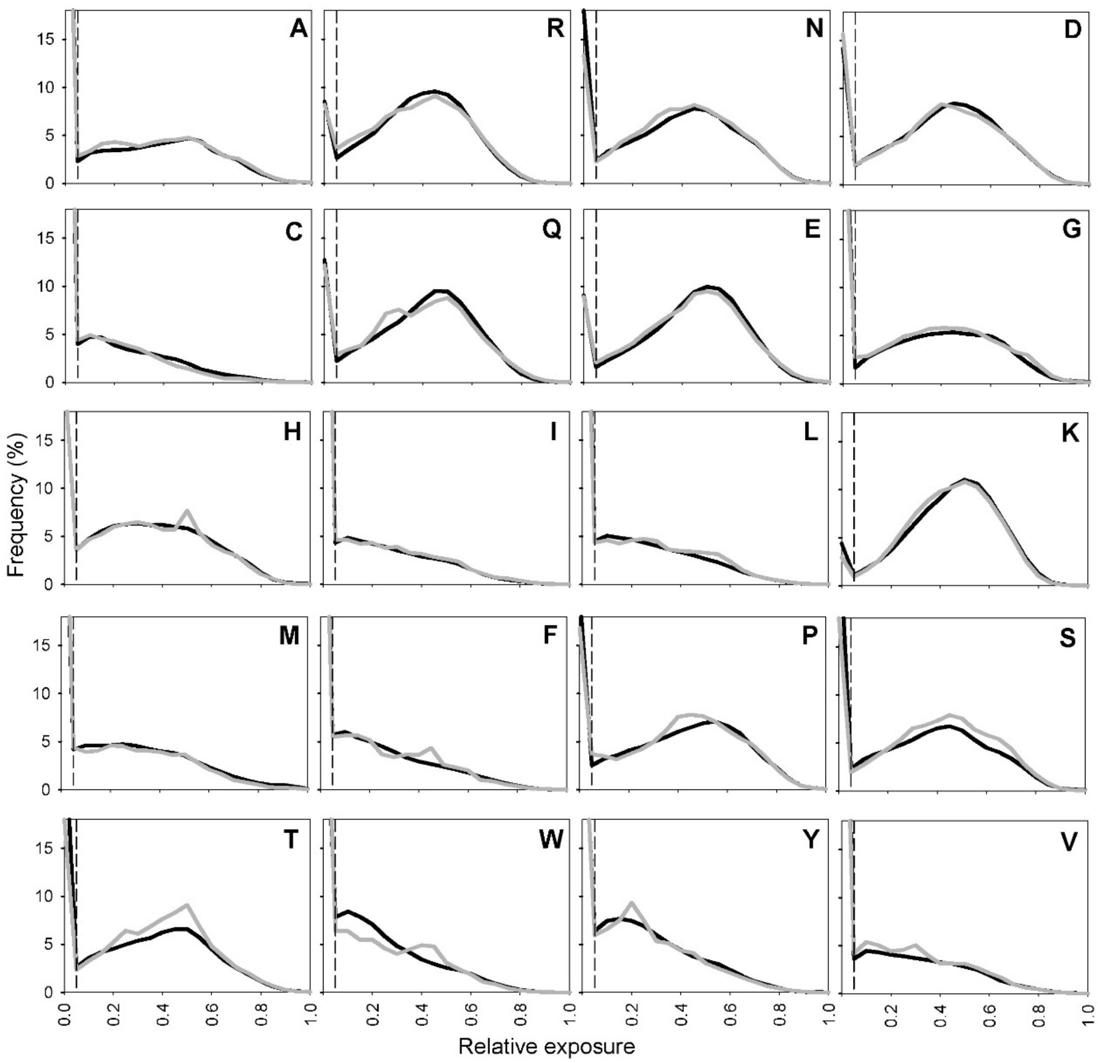

Figure S1. Relative exposure curves for the 20 amino acid residues using a 3 Å radius probe. Curves for residues belonging to non secretory-pathway (NSP) and secretory pathway (SP) proteins are in black and gray, respectively. Dashed lines indicate the cut off value of relative exposure (5%) below which a residue is considered buried.

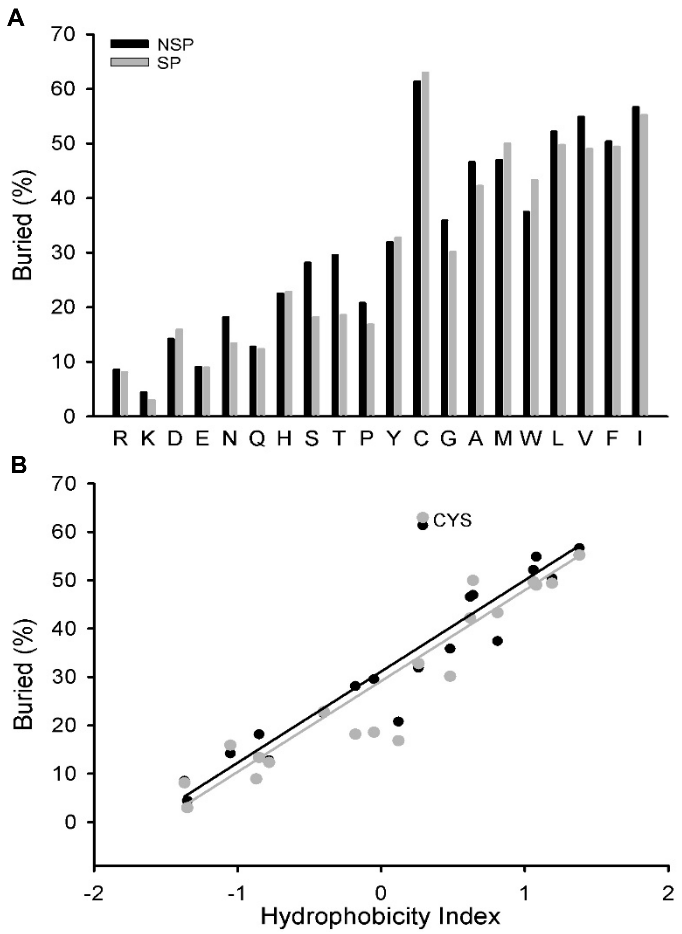

Figure S2. The proportion of buried residues is similar for secretory and non-secretory pathway proteins. (A) Proportion of buried residues in NSP (black bars) and SP (gray bars) proteins. These values are the frequency of residues corresponding to zero relative exposure shown in Figure 3-figure supplement 1. **(B)** Correlation between the proportion of hidden residues and the hydrophobicity index of Fauchere-Pliska (octanol-water partition constant)[1].

Table S1. Buried Asn in the tripeptides Asn-X-Ser and Asn-X-Thr in SP proteins. Factors that impair glycosylation by OST are highlighted in red: specific residues at +1 and +3, distances to C-terminal end lower than 60 residues, and short distances to transmembrane segments.

	PDB_chain	UNIPROT	Position	Sequon	Distance to C-terminal	Distance to TM
**Ser at +2**						
	1ck7_A	P08253	573	NWSK	87	
	1eg1_A	P07981	164	NGSL	295	
	1eg1_A	P07981	208	NTSH	251	
	1evu_A	P00488	542	NNSH	190	
	1evu_A	P00488	18	NNSN	714	
	1gw0_A	Q70KY3	281	NTST	342	
	1h0p_A	P52013	133	NGSQ	71	
	1kqr_A	P12473	132	NASQ	644	
	1ll1_A	P04253	449	NNSA	179	
	1rmg_A	Q00001	235	NWSG	205	
	1tzl_A	Q8J136	151	NLSG	472	
	1uey_A	Q92823	716	NYSF	588	
	2i2s_A	P0C6Y8	132	NTSQ	644	
	2l3o_A	P01586	77	NLSK	89	
	2npl_X	P78310	201	NASS	164	
	2wr0_A	D0VWP8	180	NTSG	329	
	2x2h_A	Q9STC1	492	NRSV	596	
	2x2h_A	Q9STC1	342	NVSQ	746	
	2x2h_A	Q9STC1	734	NMSC	354	
	3a64_A	B7X9Z2	253	NVSN	150	
	3d66_A	Q96IY4	241	NRSF	182	
	3dgv_A	Q2KIG3	241	NRSL	182	
	3ich_A	P23284	148	NGSQ	68	
	3ixv_A	P80476	446	NNSG	180	
	3pps_A	F6N9E7	272	NTST	332	
	1sqj_A	Q8J0D2	754	NYSG	**58**	
	2d39_A	O00602	305	NWSA	**21**	
	2isn_A	A8D1K3	486	NISV	**52**	
	2v3d_A	P04062	501	NRSS	**35**	
	3q31_A	Q2TWF5	226	NLST	**48**	
	2z73_A	P31356	185	NCSF	263	**10**
	2ks9_A	P25103	18	NTSE	389	**13**
	1gzm_A	P02699	200	NESF	148	**2**
	2dwr_A	P11193	132	NDSN	643	
	1shn_A	Q9BHT8	163	NDSD	312	
	1ie4_A	P02767	118	NDSG	**29**	
	1tfp_A	P27731	121	NDSG	**29**	
	2qpf_A	P07309	118	NDSG	**29**	
	3qml_C	Q08199	233	NDSN	188	
	1h1n_A	Q8TG26	40	NESG	295	
	1iaz_A	P61914	74	NESG	140	
	2aen_A	P11196	132	NNSD	643	
	2atm_A	P49370	187	NMSP	144	
	2xkl_A	Q9Z1R3	150	NRSP	**40**	
	3i6s_A	O82777	327	NGSP	434	
Pro at +1	1dhk_B	P02873	175	NPST	71	
Pro at +1	1hwm_B	Q9AVR2	398	NPSS	166	
Pro at +1	1kdg_A	Q01738	535	NPSI	238	
Pro at +1	2bb5_A	P20062	51	NPSI	376	
Pro at +1	2bb6_A	Q9XSC9	51	NPSI	381	
Pro at +1	2cnj_D	P11717	1520	NPST	971	
Pro at +1	2id4_A	P13134	146	NPSF	668	
Pro at +1	2pmv_A	P27352	54	NPSI	363	

Pro at +1	2z73_A	P31356	14	NPSI	434	
Pro at +1	3c9z_A	P33183	397	NPSS	166	
Pro at +1	3j0a_A	O60602	472	NPSL	386	
N-glycosylated	2i6q_A	Q53HP3	447	NMSA	285	
**Thr at +2**						
	1s2b_A	P15369	161	NLTT	99	
	1sli_A	Q27701	232	NVTL	530	
	1y43_B	P24665	190	NLTT	92	
	2feb_A	P08519	3969	NLTR	579	
	2x2h_A	Q9STC1	716	NSTT	372	
	2x2h_A	Q9STC1	509	NITC	579	
	3og2_A	Q70SY0	810	NWTT	213	
	1oc5_A	Q9C1S9	431	NGTS	**45**	
	2cl2_A	Q874E3	270	NLTF	**48**	
	3kdp_B	P05027	265	NLTM	**38**	
	2ks9_A	P25103	89	NFTY	318	**2**
	1eub_A	P45452	124	NYTP	347	
	1fcq_A	Q08169	223	NLTP	159	
Pro at +1	1bvp_1	P69361	235	NPTQ	114	
Pro at +1	1lnl_A	P83040	267	NPTT	146	
Pro at +1	1n9e_A	Q96X16	464	NPTL	323	
Pro at +1	1orv_A	P22411	263	NPTV	503	
Pro at +1	1tzl_A	Q8J136	593	NPTL	**30**	
Pro at +1	2gbc_A	P14740	261	NPTV	506	
Pro at +1	2kgl_A	Q9ERE7	114	NPTE	110	
Pro at +1	2qt9_A	P27487	263	NPTV	503	
Pro at +1	2xcy_A	Q4WQS0	124	NPTP	282	
Pro at +1	3nsm_A	Q06GJ0	335	NPTK	259	
N-glycosylated	1gw0_A	Q70KY3	88	NGTS	485	
N-glycosylated	1gw0_A	Q70KY3	289	NVTF	284	
N-glycosylated	1nd5_A	P15309	301	NETQ	53	
N-glycosylated	1umz_A	Q8GZD5	93	NRTG	179	
N-glycosylated	2v5r_A	Q0E9K4	18	NSTG	1978	
N-glycosylated	3pps_A	F6N9E7	89	NGTS	475	
N-glycosylated	3pps_A	F6N9E7	290	NVTF	274	
N-glycosylated	3qwq_A	P00533	328	NATN	858	

Table S2. Buried Asn in the tripeptides Asn-X-Ser and Asn-X-Thr at the protein-protein interfaces of SP proteins. Residues at +1 and +3 sites that are suboptimal, and distances to C-terminal end lower than 60 residues are highlighted in red.

	PDB_chain	UNIPROT	Position	Sequon	Distance to C-terminal	Comments
**Ser at +2**						
	2gk2_A	P13688	104	NASL	422	
	1osg_A	Q9Y275	242	NNSC	**43**	
	1tzl_A	Q8J136	140	NGSN	483	
	1a0j_A	P35033	138	NLSG	100	
	3k6s_A	P20702	1050	NLSF	113	
	1scf_A	P21583	97	NYSI	176	
	2o26_A	P20826	97	NYSI	176	
	2id5_A	Q96FE5	505	N**D**SM	115	
Pro at +1	1cf8_H	A2NUE8	61	N**P**SL	157	
Pro at +1	1ezv_X		61*	N**P**SL	66*	Non physiological immunocomplex
Pro at +1	1i9r_H		52*	N**P**SN	167*	Non physiological immunocomplex
Pro at +1	1xu7_A	P28845	270	N**P**SR	**22**	
Pro at +1	3uaj_H		60*	N**P**SL	176*	Non physiological immunocomplex
**Thr at +2**						
	1cn1_A	P02866	118*	NSTH	119*	Sequon generated after post-translational polypeptide ligation
	1dqt_A	P09793	113	NLTI	110	
	1t8u_A	Q9Y663	344	NKTK	**62**	
	1xed_A	P01833	83	NLTN	681	
	1zgl_P		53*	N**E**TQ	196*	Non natural quimeric protein
Pro at +1	1akj_D	P01732	49	N**P**TS	186	
Pro at +1	1kdg_A	Q01738	510	N**P**TA	263	

*derived from the sequence in the PDB file

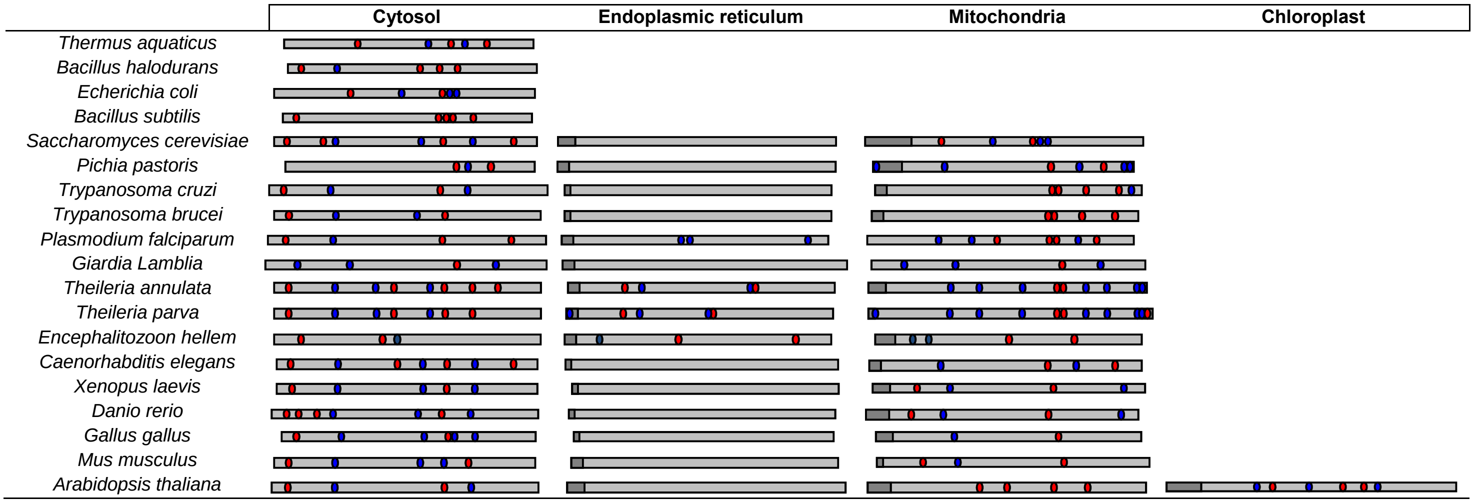

Figure S3. Location of potential sequons in Hsp70s in diverse cellular compartments from selected organisms.

The positions of sequons Asn-X-Ser and Asn-X-Thr are indicated as blue and red circles, respectively. Export signal and transit peptides are marked in dark gray.

```
SSA1      -------------------------------------------------MSKAVGIDLGTT 12
SSA4      -------------------------------------------------MSKAVGIDLGTT 12
KAR2      MFFNRLSAGKLLVPLSVVLYALFVVILPLQNSFHSSNVLVRGADDVENYGTVIGIDLGTT 60
                                                           ...:*******

SSA1      YSCVAHFANDRVDIIANDQGNRTTPSFVAFTDTERLIGDAAKNQAAMNPSNTVFDAKRLI 72
SSA4      YSCVAHFANDRVEIIANDQGNRTTPSYVAFTDTERLIGDAAKNQAAMNPHNTVFDAKRLI 72
KAR2      YSCVAVMKNGKTEILANEQGNRITPSYVAFTDDERLIGDAAKNQVAANPQNTIFDIKRLI 120
          ***** : *.:.:*:**:**** ***:***** ***********.* ** **:** ****

SSA1      GRNFNDPEVQADMKHFPFKLIDVDGKPQIQVEFKGETKNFTPEQISSMVLGKMKETAESY 132
SSA4      GRKFDDPEVTNDAKHYPFKVIDKGGKPVVQVEYKGETKTFTPEEISSMILTKMKETAENF 132
KAR2      GLKYNDRSVQKDIKHLPFNVVNKDGKPAVEVSVKGEKKVFTPEEISGMILGKMKQIAEDY 180
          * :::* .*  * ** **:::: .*** ::*. ***.* ****:**.*:* ***: **.:

SSA1      LGAKVNDAVVTVPAYFNDSQRQATKDAGTIAGLNVLRIINEPTAAAIAYGLDKKGKEEH- 191
SSA4      LGTEVKDAVVTVPAYFNDSQRQATKDAGTIAGLNVLRIINEPTAAAIAYGLDKKSQKEHN 192
KAR2      LGTKVTHAVVTVPAYFNDAQRQATKDAGTIAGLNVLRIVNEPTAAAIAYGLDK-SDKEHQ 239
          **::*..***********:*******************:************** ..:**

SSA1      VLIFDLGGGTFDVSLLSIEDGIFEVKATAGDTHLGGEDFDNRLVNHFIQEFKRKNKKDLS 251
SSA4      VLIFDLGGGTFDVSLLSIDEGVFEVKATAGDTHLGGEDFDSRLVNFLAEEFKRKNKKDLT 252
KAR2      IIVYDLGGGTFDVSLLSIENGVFEVQATSGDTHLGGEDFDYKIVRQLIKAFKKKHGIDVS 299
          ::::**************::*:***:**:*********** ::*. : : **:*:  *::

SSA1      TNQRALRRLRTACERAKRTLSSSAQTSVEIDSLFEGIDFYTSITRARFEELCADLFRSTL 311
SSA4      TNQRSLRRLRTAAERAKRTLSSSAQTSIEIDSLFEGIDFYTSITRARFEELCADLFRSTL 312
KAR2      DNNKALAKLKREAEKAKRALSSQMSTRIEIDSFVDGIDLSETLTRAKFEELNLDLFKKTL 359
           *:::* :*:  .*:***:***. .* :****:.:***:  ::***:****  ***:.**

SSA1      DPVEKVLRDAKLDKSQVDEIVLVGGSTRIPKVQKLVTDYFNGKEPNRSINPDEAVAYGAA 371
SSA4      EPVEKVLADSKLDKSQIDEIVLVGGSTRIPKVQKLVSDFFNGKEPNRSINPDEAVAYGAA 372
KAR2      KPVEKVLQDSGLEKKDVDDIVLVGGSTRIPKVQQLLESYFDGKKASKGINPDEAVAYGAA 419
          .****** *: *:*.::*:**************:*: .:*:**:..:.************

SSA1      VQAAILTGDESSKTQDLLLLDVAPLSLGIETAGGVMTKLIPRNSTIPTKKSEIFSTYADN 431
SSA4      VQAAILTGDQSSTTQDLLLLDVAPLSLGIETAGGIMTKLIPRNSTIPTKKSEVFSTYADN 432
KAR2      VQAGVLSGEEG--VEDIVLLDVNALTLGIETTGGVMTPLIKRNTAIPTKKSQIFSTAVDN 477
          ***.:*:*::.  .:*::**** .*:*****:**:** ** **::******::*** .**

SSA1      QPGVLIQVFEGERAKTKDNNLLGKFELSGIPPAPRGVPQIEVTFDVDSNGILNVSAVEKG 491
SSA4      QPGVLIQVFEGERTRTKDNNLLGKFELSGIPPAPRGVPQIEVTFDIDANGILNVSAVEKG 492
KAR2      QPTVMIKVYEGERAMSKDNNLLGKFELTGIPPAPRGVPQIEVTFALDANGILKVSATDKG 537
          ** *:*:*:****: :***********:**************** :*:****:***.:**

SSA1      TGKSNKITITNDKGRLSKEDIEKMVAEAEKFKEEDEKESQRIASKNQLESIAYSLKNTIS 551
SSA4      TGKSNKITITNDKGRLSKEDIDKMVAEAEKFKAEDEQEAQRVQAKNQLESYAFTLKNSVS 552
KAR2      TGKSESITITNDKGRLTQEEIDRMVEEAEKFASEDASIKAKVESRNKLENYAHSLKNQVN 597
          ****:.**********::*:*::** *****  ** .   :: ::*:**. *.:*** :.

SSA1      EAG--DKLEQADKDTVTKKAEETISWLDSN-TTASKEEFDDKLKELQDIANPIMSKLY-Q 607
SSA4      ENNFKEKVGEEDARKLEAAAQDAINWLDAS-QAASTEEYKERQKELEGVANPIMSKFYGA 611
KAR2      GDLG-EKLEEEDKETLLDAANDVLEWLDDNFETAIAEDFDEKFESLSKVAYPITSKLY-- 654
               :*: : *  .:   *::.:.*** .  :*  *::.:: :.*. :* ** **:*

SSA1      AGGAPGGAAGGAPGGFPGGAPPAPEAEGPTVEEVD- 642
SSA4      AGGAPG--AGPVPG--AGAGPTGAPDNGPTVEEVD- 642
KAR2      -GGADGSGAADYDD-------EDEDDDGDYFEHDEL 682
           *** *  *.   .            :*  .*. :
```

Figure S4. Sequence alignment of *Saccharomyces cerevisiae* Hsp70s. Sequons in the cytosolic homologues Ssa1 and Ssa4 are indicated in yellow. Alignment was performed with CLUSTALW[2].

Table S3. Sequons in the cytosolic Hsp70s Ssa1 and Ssa4, their aligned sequence in Kar2 and the sequons introduced in Kar2.

Mutant	Kar2	Ssa1	Ssa4	Sequon
1	80-GNRIT	32-GNRTT	32-GNRTT	80-GNRTT
2	158-KVFTP	110-KNFTP	110-KTFTP	158-KNFTP
3	196-FNDAQ	148-FNDSQ	148-FNDSQ	196-FNDTQ
4	404-ASKGI	356-PNRSI	357-PNRSI	404-ANKTI
5	459-RNTAI	413-RNSTI	414-RNSTI	459-RNTTI
6	529-LKVSA	483-LNVSA	484-LNVSA	529-LNVTA
7	625-DNFET	578-SNTTA	581-ASQAA	625-DNFTT

Table S4. Primers used for BiP *N*-glycosylation study.

Name	Sequence 5' to 3'	Reference
JJC228	GTGAAGTTCGAGAACGACACCCTGGTGAAC	This study
JJC229	GTTCACCAGGGTGTCGTTCTCGAACTTCAC	This study
JJC230	GTGAAGTTCGAGAACGACTCCCTGGTGAAC	This study
JJC231	GTTCACCAGGGAGTCGTTCTCGAACTTCAC	This study
JJC232	CATCGACTTCAACGAGACCGGCAACATCCTG	This study
JJC233	CAGGATGTTGCCGGTCTCGTTGAAGTCGATG	This study
JJC234	CATCGACTTCAACGAGTCCGGCAACATCCTG	This study
JJC235	CATCGACTTCAACGAGTCCGGCAACATCCTG	This study
JJC236	GAACACCCCCATCAACGACACCCCCGTGCTG	This study
JJC237	CAGCACGGGGGTGTCGTTGATGGGGGTGTTC	This study
JJC238	GAACACCCCCATCAACGACTCCCCCGTGCTG	This study
JJC239	CAGCACGGGGGAGTCGTTGATGGGGGTGTTC	This study
JJC340	GGCAACAACAAGACCCGCGC	This study
JJC341	GCGCGGGTCTTGTTGTTGCC	This study
JJC344	CTGGTGAACCGCACCGAGCTG	This study
JJC345	CAGCTCGGTGCGGTTCACCAG	This study
LZBS15	AAGGAATGGATCCCATGAACTCAGCATGTGCTACTCC	This study
LZBS16	AAGGAATGAGCTCAATTGCATTTGCGCAATAGAGC	This study
LZBS20	ttccagattacgctCACGACGAATTGTAGATAAAATAG	This study
LZBS21	catcgtatgggtaTTCGAAATAATCACCATCGTC	This study
JJC558	GCTAATGAGCAAGGTAACAGAactACCCCATCTTACGTGGCATTCACC	This study
JJC559	GGTGAATGCCACGTAAGATGGGGTagtTCTGTTACCTTGCTCATTAGC	This study
JJC562	CCTGCTTATTTCAATGACactCAAAGACAAGCCACCAAGG	This study
JJC563	CCTTGGTGGCTTGTCTTTGagtGTCATTGAAATAAGCAGG	This study
JJC564	CATACTTTGATGGTAAGAAGGCCaacAAGactATTAACCCAGATGAAGC	This study
JJC565	GCTTCATCTGGGTTAATagtCTTgttGGCCTTCTTACCATCAAAGTATG	This study
JJC566	CTCCATTAATTAAGAGAAATACTactATTCCTACAAAGAAATCCC	This study
JJC567	GGGATTTCTTTGTAGGAATagtAGTATTTCTCTTAATTAATGGAG	This study
JJC568	GACGCTAATGGTATTCTGaatGTGactGCCACAGATAAGGGAACTG	This study
JJC569	CAGTTCCCTTATCTGTGGCagtCACattCAGAATACCATTAGCGTC	This study
JJC570	GGTTAGATGATAACTTTacaACCGCCATTGCTGAAGACTTTG	This study
JJC571	CAAAGTCTTCAGCAATGGCGGTtgtAAAGTTATCATCTAACC	This study
JJC643	CTTCTTTTCTCCTTTGACACTTACTTCTACAGC	This study
JJC644	aatTTTACTCCAGAAGAAATTTCTGGTATGATCTTGG	This study
LZBS26	GCTAATGAGCAAGGTcaaAGAactACCCCATCTTACGTGGCATTCACC	This study
LZBS27	GGTGAATGCCACGTAAGATGGGGTagtTCTttgACCTTGCTCATTAGC	This study
LZBS28	CCTGCTTATTTCcaaGACactCAAAGACAAGCCACCAAGG	This study
LZBS29	CCTTGGTGGCTTGTCTTTGagtGTCttgGAAATAAGCAGG	This study
LZBS30	CTCCATTAATTAAGAGAcaaACTactATTCCTACAAAGAAATCCC	This study
LZBS31	GGGATTTCTTTGTAGGAATagtAGTttgTCTCTTAATTAATGGAG	This study

Underlined are the restriction sites incorporated in the primers.

Table S5. Plasmids used for BiP *N*-glycosylation study.

Name	Description	Reference
pRS416		3
Ec316	pRS416-*KAR2*	This study
Ec317	pRS416-*KAR2*-HA	This study
Ec318	pRS416-*KAR2*-A462T-HA	This study
Ec319	pRS416-*KAR2*-K530N, S532T-HA	This study
Ec320	pRS416-*KAR2*-E628T-HA	This study
Ec321	pRS416-*KAR2*-A199T-HA	This study
Ec326	pRS416-*KAR2*-S405N, G407T-HA	This study
Ec330	pRS416-*KAR2*-V159N-HA	This study
Ec335	pRS416-*KAR2*-I83T-HA	This study
Ec381	pRS416-*KAR2*-N81Q-I83T-HA	This study
Ec382	pRS416-*KAR2*-N197Q-A199T-HA	This study
Ec383	pRS416-*KAR2*-N460Q-A462T-HA	This study

Table S6. Yeast strains used for BiP *N*-glycosylation study.

Name	Genotype	Reference
KAR2	*MAT*a *leu2-3,112 ura3-52 ade2-101*	4
kar2-159	*MAT*a *leu2-3,112 ura3-52 ade2-101 kar2-159*	5

Figure S5. Original gels shown in Fig. 2C

.

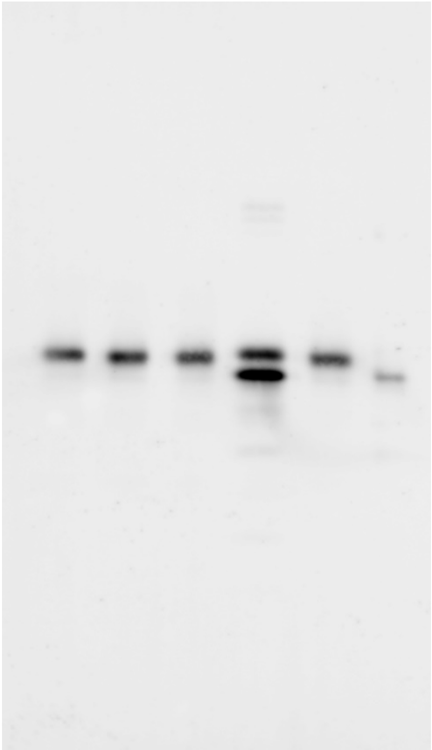

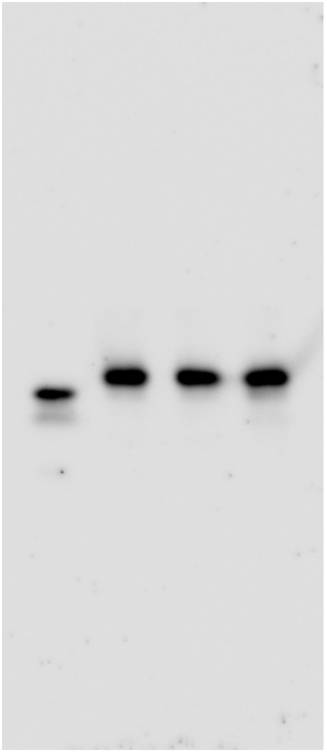

Supplementary file 1

Classification between SP and NSP of PDB structures employed in this study.

Supplementary file 2

Buried sequons in gains of *N*-glycosylation mutations associated with human genetic diseases.

References

1. Fauchere, J. L., Charton, M., Kier, L. B., Verloop, A. & Pliska, V. Amino acid side chain parameters for correlation studies in biology and pharmacology. *Int. J. Pept. Prot. Res.* **32,** 269-278 (1988).
2. Thompson, J. D., Higgins, D. G. & Gibson, T. J. CLUSTAL W: improving the sensitivity of progressive multiple sequence alignment through sequence weighting, position-specific gap penalties and weight matrix choice. *Nucl. Acids Res.* **22,** 4673-4680 (1994).
3. Mumberg, D., Muller, R. & Funk, M. Yeast vectors for the controlled expression of heterologous proteins in different genetic backgrounds. *Gene* **156,** 119-122 (1995).
4. Polaina, J. & Conde, J. Genes involved in the control of nuclear fusion during the sexual cycle of *Saccharomyces cerevisiae*. *Mol. Gen. Genetics* **186,** 253-258 (1982).
5. Scidmore, M. A., Okamura, H. H. & Rose, M. D. Genetic interactions between KAR2 and SEC63, encoding eukaryotic homologues of DnaK and DnaJ in the endoplasmic reticulum. *Mol. Biol. Cell* **4,** 1145-1159 (1993).
